# State of the Art in Neuromodulation or Spinal Cord Stimulation Therapy

**DOI:** 10.3390/jcm15176574

**Published:** 2026-08-26

**Authors:** Nafay Abdul, Milan Patel, Rohit Aiyer, Manuel Lomeli, Kalvin Chen, Alan D. Kaye, Giuliano Lo Bianco, Alaa Abd-Elsayed

**Affiliations:** 1 Department of Medicine, New York Institute of Technology College of Osteopathic Medicine at Arkansas State University, Jonesboro, AR 72401, USA; 2 Mind and Brain Link, Manhattan Beach, CA 90266, USA; 3 Department of Anesthesiology, School of Medicine and Public Health, University of Wisconsin, Madison, WI 53706, USAkjchen7@wisc.edu (K.C.); 4 Department of Anesthesiology, Louisiana State University Health Sciences Center, Shreveport, LA 71103, USA; 5 Anesthesiology and Pain Department, Fondazione Istituto G. Giglio Cefalù, Palermo, 90015 Cefalù, Italy

**Keywords:** spinal cord stimulation, neuromodulation, chronic pain, closed-loop stimulation, differential target multiplexed stimulation, loss of efficacy, artificial intelligence, opioid-sparing outcomes

## Abstract

Chronic pain continues to be a major global health burden and is frequently refractory to conventional pharmacologic and conservative therapies. Spinal cord stimulation (SCS) has emerged as an important neuromodulatory treatment for selected patients with chronic neuropathic and mixed pain syndromes. Since its introduction in the 1960s, SCS has evolved from paresthesia-based tonic stimulation into more adaptive and personalized neuromodulation. This review summarizes the current evidence regarding the mechanisms, clinical applications, technological advances, and future directions of SCS therapy. Mechanistically, SCS modulates nociceptive transmission through dorsal column and dorsal horn pathways, inhibitory neurotransmitter systems, wide-dynamic-range neuronal activity, and supraspinal pain-processing networks. Technological advances have expanded available stimulation paradigms, including burst stimulation, high-frequency stimulation, closed-loop evoked compound action potential-controlled systems, and differential target multiplexed stimulation. These approaches aim to improve analgesic durability, reduce the burden of paresthesia, and address mechanisms such as neuroinflammation and neural habituation. Clinically, SCS is used for conditions including failed back surgery syndrome, complex regional pain syndrome, painful diabetic neuropathy, ischemic limb pain, and emerging non-traditional pain states. However, outcomes remain variable and are influenced by psychological readiness, pain phenotype, anatomic factors, trial response, neurophysiologic markers, and patient engagement. Complications such as lead migration, infection, implantable pulse generator malfunction, and loss of efficacy remain important considerations. Future progress in SCS will likely depend on artificial intelligence, remote monitoring, biomarker-guided programming, and integration with multidisciplinary chronic pain care.

## 1. Introduction

Chronic pain exerts an enormous personal and economic burden, affecting more than 30% of people worldwide. Some consider chronic pain to be a disease with treatments, and it can also come with psychological impacts on patients. There are many different categories of chronic pain, such as nociceptive, neuropathic, or nociplastic, all of which require attention and treatment. In relation to these conditions, quality of life may decrease; however, with adequate pain management, these effects may reverse [1]. Low back pain is currently the leading cause of disability in most countries, and it is expected that the burden, as well as related costs, will increase in the coming decades. In 2020, low back pain affected 619 million people globally, with the number predicted to reach 843 million by 2050 [2]. Low back pain is most prevalent among women and the elderly population aged 75 and above. Chronic pain can be classed as pain that lasts over 3 months and can disrupt daily activities [3]. This pain can impact patients’ quality of life and daily activities, so treatment for low back pain is warranted.

Currently, pharmacotherapy for chronic low back pain includes NSAIDs, opioids, muscle relaxants, acetaminophen, and antidepressants, although their efficacy and safety vary [4,5,6,7,8]. Most medication classes provide limited improvement in chronic low back pain, while some treatments, such as duloxetine, have demonstrated more modest effects [8]. Long-term opioid therapy is particularly limited by variable long-term efficacy and risks of adverse effects, addiction, and overdose [6,9]. These limitations highlight the need for alternative approaches to the management of chronic low back pain.

Initially reported by Shealy et al., spinal cord stimulation delivers electrical impulses to the epidural space above the dorsal columns to interfere with nociceptive signal transmission and reduce long-term pain [10,11,12]. In patients who successfully pass a temporary trial phase, SCS yields much greater pain relief and overall functional improvement, specifically chronic back pain and leg pain depicted in a study done by Huygen et al., than standard medical management practices [12,13]. Historically, conventional SCS relied on low-frequency, high-pulse-width, and high-amplitude stimulation that induced paresthesias via the activation of large A-beta fibers [14]. However, long-term efficacy requires further investigation as it is variable across waveform types, but with advancements in programming of SCS devices, such as four adjustable parameters and an increased number of electrodes available, SCS systems can become more personalized for the patient [15,16,17]. Additionally, they have adapted a closed-loop system that continuously modulates stimulation intensity, enabling more stability and better pain management, unlike open-looped systems that are subject to failure [18].

Scope and Objective: This comprehensive review synthesizes recent advancements in neuromodulation with a heavy emphasis on spinal cord stimulation mechanism, waveform evolution, and prediction of results. This review aims to evaluate the technical refinement of SCS and highlight strategies to further optimize SCS’s long-term effectiveness while finding gaps in the current literature. Specifically, we aim to argue that spinal cord stimulation is fundamentally evolving from paresthesia-based symptom suppression toward mechanism-informed and personalized neuromodulation, a very vital transition to improve the consistency and precision of the therapeutic effects of SCS in chronic pain management.

## 2. Mechanisms of Neurostimulation

### Neurophysiology Basics

Gate control theory plays a critical role in spinal cord neurostimulation (SCS). The theory by Melzack & Wall posits that the activation of dorsal column fibers stimulate inhibitory interneurons in the dorsal horn that release gamma-aminobutyric acid (GABA) and glycine, which closes the neurological “gate” and blocks pain signal transmission, along with A-gamma and C fibers to the brain [19]. SCS leverages this mechanism by recruiting large-diameter Aβ fibers in the dorsal column, thereby activating inhibitory interneurons in Rexed Lamina II, which ultimately closes the gate to nociceptive C-fiber input. A study by Chakravarthy et al. (2019) [20] found that burst signal cord stimulation decreased wide-dynamic-range (WDR) neuronal activity in the dorsal horn, with higher burst charges yielding greater WDR suppression. Conversely, tonic SCS exhibited a relative lack of GABA receptor activation, underscoring the necessity of GABA pathways for optimal pain inhibition [19,20]. Patients with chronic back pain suffer from irregular neuromodulatory signaling, including reduced serotonin, noradrenaline, and GABA activity, which disrupts natural filtering; as such, SCS targets these pathways to restore inhibitory control [21].

## 3. Evolution of Stimulation Waveforms

Since the introduction of spinal cord stimulation (SCS) in the late 1960s, considerable advances in stimulation technology have been made to improve pain relief, reduce adverse effects, and enhance long-term efficacy. While early SCS systems relied on conventional tonic stimulation, advances in pain neurophysiology have led to the development of burst stimulation, high-frequency stimulation, closed-loop systems, and differential target multiplexed (DTM) stimulation, each designed to improve patient outcomes and address limitations of earlier technologies [22].

### 3.1. Traditional Tonic Stimulation

Conventional tonic stimulation delivers electrical stimulation at frequencies between 50 and 60 Hz, producing paresthesia by activating large-diameter Aβ fibers in the dorsal columns [23]. According to the gate control theory, activation of these fibers inhibits nociceptive transmission in the dorsal horn, thereby reducing pain perception [23,24]. Although tonic stimulation remains effective for many neuropathic pain conditions, limitations such as inconsistent paresthesia coverage, frequent programming adjustments, uncomfortable paresthesias, and loss of efficacy over time prompted the development of newer stimulation paradigms [25,26].

### 3.2. Burst Stimulation

Burst stimulation was developed to more closely mimic the natural burst-firing patterns of thalamocortical neurons involved in pain processing [27]. Unlike tonic stimulation, burst stimulation delivers clusters of pulses separated by brief pauses and can provide pain relief with little or no paresthesia. Clinical evidence from SUNBURST and PROCO, as mentioned in the cited articles, is randomized controlled trials demonstrating that burst stimulation provides analgesia comparable to or better than conventional stimulation while improving patient comfort and preference [28,29]. However, burst stimulation shows higher overall energy delivery per second than low-duty tonic stimulation, which can increase battery drain in implantable pulse generators (IPGs) or require microdosing strategies, alongside a persistent rate of non-responders in clinical practice [30].

### 3.3. High-Frequency Stimulation

High-frequency spinal cord stimulation (10 kHz), or HF10 therapy, delivers stimulation without producing paresthesia and has become one of the most significant advances in modern neuromodulation [31]. Although its precise mechanism remains incompletely understood, high-frequency stimulation appears to modulate pain pathways beyond simple dorsal column activation. The landmark SENZA-RCT demonstrated superior pain relief for both back and leg pain compared with conventional low-frequency stimulation, contributing to the widespread adoption of HF10 therapy [31]. Despite these advantages, the high charge delivery of 10 kHz stimulation demands significantly greater energy consumption, requiring patients to undergo frequent (often daily) device recharging and precluding the use of non-rechargeable IPGs. Furthermore, a non-negligible subset of patients experience primary non-response or late loss of efficacy over long-term follow-up [30].

### 3.4. Closed-Loop Stimulation

Traditional SCS systems use an open-loop design in which stimulation output remains constant despite changes in posture or lead position. Closed-loop systems overcome this limitation by continuously monitoring evoked compound action potentials (ECAPs) and automatically adjusting stimulation intensity to maintain consistent spinal cord activation [32,33]. The EVOKE randomized clinical trial demonstrated superior and more durable pain relief with ECAP-controlled closed-loop stimulation compared with conventional open-loop systems, highlighting the potential of physiologic feedback to improve long-term outcomes [34,35]. Furthermore, closed-loop technology introduces greater clinical and programming complexity, requiring precise signal-sensing calibration, artifact management, and specialized clinician expertise, alongside higher baseline device acquisition costs [30].

### 3.5. Novel Waveforms and Adaptive Stimulation

Emerging waveform strategies continue to expand SCS’s capabilities. Differential Target Multiplexed (DTM) stimulation delivers multiple electrical signals simultaneously to modulate neuronal and glial cell activity, potentially addressing neuroinflammatory mechanisms underlying chronic pain [36,37]. In a study conducted by White et al., recent randomized evidence comparing DTM SCS with conventional SCS in patients with chronic refractory axial low back pain who were not eligible for spine surgery further supports the clinical relevance of this waveform strategy, solidifying the promising results of DTM stimulation [38]. Other adaptive programming strategies allow clinicians to alternate between waveforms or adjust stimulation parameters according to patient-specific needs, representing an important step toward personalized neuromodulation. However, multiplexed and adaptive paradigms place higher demands on device processing and battery capacity while requiring increased clinical oversight to navigate extensive parameter combinations.

### 3.6. Comparative Efficacy and Limitations

Despite substantial advances in waveform technology, no single stimulation paradigm is universally superior. Treatment response continues to depend on patient selection, pain etiology, and individual neurophysiology [39]. While newer waveforms may improve patient comfort and therapeutic consistency, they introduce key trade-offs across financial, technical, and biological domains. High-frequency (10 kHz), burst, and DTM waveforms significantly increase power demands, leading to faster battery depletion, shorter non-rechargeable IPG lifespans, or increased patient burden from frequent recharging schedules [30]. Furthermore, closed-loop and multiplexed systems incur higher initial equipment costs and demand considerable programming complexity from clinicians to optimize parameters. Finally, primary non-response rates (ranging from 15% to 30% depending on modality) and long-term habituation remain constant challenges across all modern SCS waveforms, as described in Table 1, depicting that waveform choice alone cannot stop treatment from failing.

## 4. Clinical Indications and Therapeutic Targets

As mentioned previously, spinal cord stimulation (SCS) has an expanded role in managing chronic pain syndromes. It has several well-researched, established indications approved by the United States Food and Drug Administration (FDA). In the past, SCS was used for conditions related to neuropathic or mixed nociceptive–neuropathic pain that did not respond adequately to conservative therapies. Among the few widely accepted and common indications are Failed Back Surgery Syndrome (FBSS), complex regional pain syndrome (CRPS), post-laminectomy pain syndrome, and chronic neuropathic leg pain [40]. These conditions have several pathophysiological features, including central sensitization, altered dorsal horn processing, and maladaptive neuroplasticity, that enable them to respond well to neuromodulatory interventions.

Failed back surgery syndrome remains one of the most common indications for SCS implantation. Patients with FBSS often experience persistent axial low back pain with or without other radicular symptoms despite anatomically successful surgical intervention. Randomized evidence shows superior pain relief with SCS added to conventional medical management compared with conventional medical management alone in appropriately selected FBSS patients [41]. Similarly, CRPS represents a definitive indication for neuromodulation. At the same time, the notion of targeting neuropathic pain generators has been strengthened by randomized effectiveness comparisons that support dorsal root ganglion (DRG) stimulation over conventional SCS in CRPS and causalgia (pain caused by nerve injury) [40].

In addition to classic indications, the overall therapeutic scope of SCS continues to expand. Other conditions, such as peripheral neuropathic pain like painful diabetic neuropathy, have shown clinically meaningful benefit in randomized trials when using high-frequency SCS compared to normal/conventional medical management [41]. Ischemic limb pain in selected patients with critical limb ischemia has also been shown in several randomized trials and reviews, with findings indicating potential benefit in limb salvage and overall patient clinical status in select populations [23,42,43]. However, it is important to note that heterogeneity in patient selection and trial methods remains a concern when interpreting vascular and clinical indications for these treatments [23,43].

Furthermore, emerging evidence has explored neuromodulation for non-traditional pain targets, such as visceral pain syndromes, post-stroke pain, and spinal cord injury-related pain states [44]. For instance, chronic pancreatitis-related visceral pain has been reported to improve in several clinical series. It has more recently been tested in sham-controlled paradigms, being administered in a control group alongside active treatment, which depicts promise in the further usage of SCS, but also the need for long-term evaluation [45,46]. On the other hand, central post-stroke pain currently remains under experimentation. Still, the neurosurgery-related literature shows potential benefit in select cases and highlights uncertainty regarding predictors of response in centrally mediated pain states [47]. As device capabilities evolve, DRG stimulation and hybrid SCS-DRG strategies may further improve localization and direct targeting of dermatomal pain while maintaining the ability to address axial pain generators [40].

### Spinal Cord Stimulation Pathway

According to Guy’s and St Thomas’ NHS Foundation Trust, the spinal cord stimulation treatment pathway is a plan in which patients can be evaluated to see if SCS is the right option. This plan outlines pre-implant management, the spinal cord stimulator trial, and post-stimulator outcomes. The pathway also outlines spinal cord stimulator contraindications and management directions in regard to medication usage and patient demographics [48].

Of note, according to the British Pain Society, there are special considerations for electrode implantations. According to Table 3 in the British Pain Society document, a pre-operative MRI of the spine is important for assessing the potential of spinal stenosis, as this will affect electrode placement when using a spinal cord stimulator [49].

## 5. Long-Term Efficacy and Loss of Efficacy (LoE)

While spinal cord stimulation has demonstrated short- and intermediate-term efficacy in the treatment of chronic pain conditions, there are limited data supporting improvements in long-term outcomes. Clinical experience and longitudinal studies suggest that many patients experience sustained pain relief and functional improvement. In contrast, others demonstrate diminishing therapeutic benefit over time, which is commonly referred to as loss of efficacy (LoE) [50,51]. This phenomenon is often discussed within a 1–2-year time window; however, the durability of this treatment varies across population samples, device strategies, and follow-up maintenance [50,51].

Several mechanisms have been proposed as possible causes of loss of efficacy. The two that are heavily cited in the world literature are neural adaptation and habituation, which essentially mean that there will be reduced responsiveness of spinal and supraspinal pain networks to a constant-stimulation-related strategy [31]. Structural and technical factors can also contribute; clinically significant lead migration is a recognized cause of reduced paresthesia coverage at the site of the pain and reduced pain relief [52]. Biological factors, including local tissue response and changes in impedance (the resistance of biological conduction to the flow of electrical current) around the lead–epidural interface, are also theorized to reduce the effective delivery of stimulation over time and may interact with patient movement [48].

Some technological advances and developments aimed at reducing LoE through more consistent dosing and adaptive control have become more prevalent. ECAP-controlled closed-loop SCS has demonstrated superior, more stable outcomes compared with open-loop systems over 12 months in a double-blind randomized trial, and observational follow-up suggests maintenance of benefit beyond the early post-implant period [26,48]. In addition, current clinical practice emphasizes strategies such as periodic programming, waveform cycling, and patient engagement to address evolving pain patterns and counteract habituation. To expand on periodic programming, it involves scheduled or symptom-driven adjustments of stimulation parameters, such as amplitude, pulse width, and frequency, to optimize neural target engagement. At the same time, waveform cycling refers to intentional alternation between different stimulation waveforms, such as tonic, burst, or high-frequency stimulation, to engage distinct neurophysiologic mechanisms and reduce neural habituation [26]. Some approaches are deemed as “rescue” approaches, such as temporary interruption, which have also been noted as methods to restore analgesia in specific subsets of patients with suspected habituation [53].

LoE prevention is increasingly framed as an issue rooted at the systems level. More consistent and durable outcomes are more likely when neuromodulation studies and follow-ups focus on optimizing physical function, psychological coping, and ongoing reassessment of pain generators rather than treating SCS as an endpoint for targeting chronic pain [26,54,55].

## 6. Contraindications

It is important to note that while spinal cord stimulation is a great option in some cases, there are situations where the use of SCS should be avoided. However, before spinal cord stimulation is even considered, pharmacologic management must be utilized before as an initial treatment plan. If pharmacologic management and other non-invasive treatments are ineffective, then spinal cord stimulation can be used.

Beginning with perioperative surgical procedure standards, any active infection or coagulopathy concerns are contraindications for spinal cord stimulation usage. Additionally, mental health concerns and psychiatric comorbidities such as depression and anxiety are also contraindications [56]. Furthermore, additional situations in which SCS would be a contraindication include memory or cognitive difficulties, chronic conditions such as postural tachycardia syndrome, and active substance or addiction [30].

Lastly, having a spinal cord stimulator active while operating a motor vehicle is prohibited. It should be known that while driving or operating heavy machinery, the spinal cord stimulator must be turned off [57].

## 7. Predictors of Clinical Outcome

Although spinal cord stimulation (SCS) has demonstrated clinical benefit in several chronic pain populations, outcomes remain variable among patients. Some individuals experience durable pain relief, improved function, and reduced reliance on medications, while others experience limited benefit or loss of efficacy over time [58]. Therefore, identifying predictors of clinical outcome has become an important area of research in neuromodulation. Predictors of response may enable clinicians to select candidates better, optimize treatment before implantation, and develop more personalized stimulation strategies [58]. Current evidence suggests that outcomes are influenced by a combination of psychological, physiological, anatomic, neurophysiologic, and computational factors.

### 7.1. Psychological Predictors

Psychological factors are among the strongest patient-related predictors of successful SCS outcomes [59]. Chronic pain is not only a sensory experience but also involves emotional, cognitive, and behavioral components. Depression, anxiety, pain catastrophizing, poor coping style, and unrealistic expectations have all been associated with less favorable outcomes following SCS implantation [23,60]. The PHQ-9 is a validated screening tool for depression with a cutoff score of 10. This survey can be used to help address and aid in determining if the patient presents with depression. This is important because of its effects on the overall treatment plan [32]. Additionally, the Pain Catastrophizing Scale (PCS) is widely used as a 13-item self-report measure to assess catastrophic thinking in regard to pain in adults with and without chronic pain. A cutoff score of greater than or equal to 30 points is indicative of catastrophic thinking and will be used when creating a treatment for a patient [61]. Depression may worsen pain perception, reduce motivation to participate in rehabilitation, impair adherence to follow-up care, and negatively influence patient satisfaction after implantation [60]. Similarly, catastrophizing, defined as an exaggerated negative cognitive response toward pain, has consistently been associated with increased pain intensity, disability, and poorer treatment outcomes across chronic pain populations [60].

Coping style also appears to influence clinical response. Patients who demonstrate active coping strategies, realistic goal setting, and engagement in physical rehabilitation generally achieve better functional outcomes than patients who rely primarily on passive coping or activity avoidance [23]. Expectations likewise play an important role. Patients who expect complete elimination of pain may perceive treatment as unsuccessful despite clinically meaningful improvements. In contrast, individuals with realistic expectations often report greater satisfaction with moderate pain reduction accompanied by improved mobility, sleep quality, and quality of life [23]. Because of these findings, comprehensive psychological assessment has become a routine component of preoperative SCS evaluation [23,59]. Rather than excluding patients solely because of depression or anxiety, psychological screening aims to identify modifiable risk factors that can be optimized before implantation. Untreated psychiatric illness, severe catastrophizing, active substance abuse, inadequate social support, secondary gain, and poor engagement with previous therapies have all been identified as potential predictors of poorer clinical outcomes [23,32].

### 7.2. Physiological and Anatomic Predictors

Physiological and anatomic characteristics also contribute significantly to SCS outcomes. One of the strongest predictors is the underlying pain phenotype, as SCS has consistently demonstrated greater efficacy in neuropathic and mixed neuropathic–nociceptive pain conditions, including failed back surgery syndrome (FBSS), persistent spinal pain syndrome, complex regional pain syndrome (CRPS), and painful diabetic neuropathy [59,61]. Patients with well-localized neuropathic pain generally respond better than those with diffuse, widespread, or primarily nociplastic pain because stimulation can be more precisely targeted [61]. Nerve integrity may also influence treatment response. Patients with preserved neural pathways are thought to achieve better outcomes because stimulation can effectively modulate intact pain circuits. In contrast, advanced nerve injury or severe deafferentation may reduce responsiveness to therapy [61].

Response to temporary trial stimulation remains an important clinical predictor before permanent implantation. Traditionally, achieving approximately 50% pain relief during the trial has been considered indicative of a successful candidate, although improvements in physical function, sleep quality, medication reduction, and patient satisfaction are increasingly recognized as equally important measures of success [59]. However, recent evidence suggests that trial stimulation alone does not consistently predict long-term outcomes and should be interpreted alongside psychological and clinical factors rather than as an independent predictor [62].

### 7.3. Neuroimaging and Neurophysiologic Markers

Advances in neuroimaging have improved the understanding of why some patients respond better than others to SCS. Functional magnetic resonance imaging (fMRI) studies have demonstrated that chronic pain is associated with altered connectivity within several pain-processing regions, including the anterior cingulate cortex, insula, prefrontal cortex, thalamus, and default mode network [63]. These findings suggest that baseline brain network activity may eventually serve as a biomarker for predicting treatment response.

Electroencephalography (EEG) has also emerged as a promising tool because it provides a non-invasive measure of cortical activity. Recent studies have combined EEG biomarkers with predictive algorithms to distinguish responders from non-responders before implantation [64]. In addition, evoked compound action potentials (ECAPs) are used in modern closed-loop SCS systems to monitor spinal cord activation and automatically adjust stimulation intensity, providing more consistent therapy despite changes in posture or lead position [26]. Together, these neuroimaging and neurophysiologic markers represent an important step toward more objective and personalized neuromodulation.

### 7.4. Machine Learning and Neuromodulation

Machine learning and artificial intelligence are emerging strategies for improving patient selection, predicting SCS outcomes, optimizing programming, and enhancing long-term therapeutic efficacy [30]. Traditional selection relies on clinical judgment, psychological assessment, imaging, and trial stimulation; however, these factors alone do not fully explain the variability in long-term outcomes [65,66].

Machine learning models can integrate multiple patient variables, including age, pain duration, diagnosis, opioid use, psychological measures, and functional status, to estimate the likelihood of treatment success [65]. A study conducted by Hadanny et al. focused on treatment response prediction to spinal cord stimulation using machine learning models. The algorithm had an AUC of 0.757, sensitivity of 61.7%, specificity of 80%, and an accuracy of 73.4% [30]. The cohort size was n = 79; however, further studies must be conducted to support external validation of the models tested [30]. This approach supports the growing field of precision neuromodulation, where stimulation parameters and patient selection can be tailored to an individual’s unique clinical profile. Although these predictive models remain investigational and require larger validation studies, they represent a promising direction for improving long-term SCS outcomes [66].

In summary, predictors of SCS outcome are multifactorial. Psychological readiness, pain phenotype, anatomic pain distribution, nerve integrity, trial stimulation response, neurophysiologic markers, and machine learning-based prediction models all contribute to understanding which patients are most likely to benefit. As the field advances, the goal is to move from generalized patient selection to personalized neuromodulation, enabling clinicians to predict response better, optimize stimulation parameters, and improve long-term clinical outcomes.

## 8. Opioid-Sparing and Functional Outcomes

One of the major advantages of spinal cord stimulation (SCS) is its potential to improve pain while reducing reliance on long-term opioid therapy. Given the ongoing opioid epidemic and the well-established risks associated with chronic opioid use, including tolerance, dependence, overdose, endocrine dysfunction, and opioid-induced hyperalgesia, there has been increasing interest in therapies that provide effective analgesia while minimizing opioid exposure [67]. Although opioid reduction is not universally achieved, numerous studies have demonstrated that successful SCS implantation is associated with decreased opioid consumption, particularly among patients who experience sustained pain relief and functional improvement [23,38].

The opioid-sparing effects of SCS are thought to result from its ability to modulate pain processing rather than simply masking symptoms. By inhibiting nociceptive transmission within the dorsal columns and dorsal horn while also influencing supraspinal pain-processing networks, SCS reduces pain intensity and may improve the emotional and affective components of chronic pain [64]. As pain becomes better controlled, patients often require lower doses of opioid medications, thereby reducing the risk of medication-related adverse effects while improving participation in rehabilitation and other multidisciplinary treatment strategies [67].

Beyond pain reduction, the primary goal of SCS is improvement in overall function and quality of life. Contemporary clinical trials increasingly emphasize functional outcomes rather than pain scores alone. Successful SCS therapy has been associated with improvements in physical function, sleep quality, mobility, activities of daily living, and patient-reported quality of life [38,67]. Some studies have also demonstrated improvements in work productivity and return-to-work rates among appropriately selected patients. However, these outcomes vary depending on the underlying pain condition, duration of disability, and psychosocial factors [23,54]. These findings reinforce the concept that treatment success should be measured not only by pain reduction but also by restoration of meaningful daily function.

From a health economics perspective, SCS is a cost-effective intervention for carefully selected patients despite its relatively high initial implantation cost. Although implantation and device expenses are substantial, reductions in long-term opioid use, healthcare utilization, repeat interventions, and disability-related costs may offset these expenditures over time [68]. Several economic analyses have demonstrated that SCS becomes increasingly cost-effective over longer follow-up periods when compared with continued conventional medical management, particularly in patients with refractory neuropathic pain syndromes [23,69]. Consequently, SCS should be viewed not only as a pain management intervention but also as a strategy that may improve long-term patient function while reducing the overall economic burden associated with chronic pain.

## 9. Limitations, Safety, and Complications

### 9.1. Device-Related: Lead Migration, Battery Failure, Infection

While spinal cord stimulation is an effective treatment for various chronic pain conditions, physicians as well as patients should be aware of potential complications associated with this procedure. The literature reveals that the most frequent complications are lead migration, electrode dislocation or breakage, and implantable pulse generator (IPG) failure. Biological complications reported include infection, CSF leakage, pain at the incision or electrode site, discomfort at the local implantable pulse generator site, inflammation, and fever of unknown origin. Paralysis is an infrequent (0.03%) but possible complication [70]. In order to manage a serious infection in regard to a spinal cord stimulator, the device must be explanted until the infection is controlled. However, if the infection is limited to superficial tissues, then the device may not need to be explanted. More serious complications such as oversedation, anesthesia issues, airway compromise, and anaphylactic reactions can be handled by an anesthesiologist [71].

Lead migration is the most common complication of percutaneous spinal cord stimulation, and in recent years it has been addressed by using various anchoring techniques to reduce its incidence. Migration can lead to potentially serious adverse outcomes; for example, if lead migrates anteriorly, then there may be encroachment on the spinal cord and subsequent complications. Furthermore, anterior electrode displacement may be secondary to a posterior hematoma or inadvertent misplacement during insertion; therefore, the clinician must recognize the symptoms and obtain immediate imaging to confirm migration. IPG failure has been an issue since the inception of spinal cord stimulation, with newer high-frequency systems gaining popularity to optimize pain management and decrease complications as compared to traditional systems. Rechargeable implantable pulse generators have been the preferred power source [71].

With lead fractures, broken or misplaced leads may be better detected on radiography than on CT because the leads course in and out of the CT scan plane. For evaluating electrode location, both CT and radiography (X-ray imaging) are valuable. For instance, CT is ideally suited to identify complications such as hematomas, abscesses (with IV contrast administration), impingement on the thecal sac or spinal cord, and CSF leakage (with intrathecal contrast administration). In contrast, X-ray imaging can help identify lead migration [71].

Surgical paddle lead placement has been associated with slightly higher initial postoperative complication rates than percutaneous lead placement. Still, long-term costs and reoperation rates are lower in surgical paddle lead placement cohorts [72].

More importantly, extensive research confirms that lead migration is lower in patients with paddle lead placement than with percutaneous lead placement, including a study by *Gomez-Gonzalez* (paddle: 2.94%, percutaneous: 10.83%, *p* = 0.051) [38]. Infection rates are thought to be in the range of 2.4–3.1% in patients implanted with spinal cord stimulators [73]. In one of the largest studies (over 3000 patients reviewed) that investigated SCS implants and infection, Hoelzer et al. confirmed an infection rate of (2.45%). Furthermore, this study did not show an increased rate of infection for patients who used tobacco, had diabetes, or were obese. Other conclusions that can be drawn from this study include the importance of using an occlusive dressing over the incision in the post-operative period to reduce the rate of infection, as well as the positive impact of post-operative antibiotics on infection rates [74].

### 9.2. Patient-Related: Poor Compliance, Psychological Barriers

To help reduce the risk of non-compliance with spinal cord stimulation, the multidisciplinary pain specialist team needs to educate patients and/or caregivers about the therapy process, the rationale for this treatment modality, and the potential risks and benefits. It is important to set realistic expectations for potential pain relief and to emphasize the need for patients to be actively engaged with the device regarding programming and ensuring it is functioning. Finally, a realistic timeline for improvement in a patient’s chronic pain condition should also be brought forth to the patient when discussing spinal cord stimulation as a treatment option [75].

Psychological determinants and the selection of patients appropriate for spinal cord stimulation are complex processes. A multidisciplinary European panel used the RAND/UCLA Appropriateness Method (RUAM) to assess the appropriateness of SCS for 386 clinical scenarios in four pain areas: chronic low back pain and/or leg pain, complex regional pain syndrome, neuropathic pain syndromes, and ischemic pain syndromes. In addition, the panel identified a set of psychosocial factors that are relevant to the decision for SCS treatment, which included lack of engagement, dysfunctional coping, unrealistic expectations, inadequate daily activity level, problematic social support, secondary gain, psychological distress/mental health problems, and unwillingness to reduce high-dose opioids [54].

Several factors may contribute to poor or non-compliance with spinal cord stimulation; therefore, a comprehensive history and review of the patient’s compliance with non-spinal cord stimulator treatments should be conducted. If patients are non-engaged with prior treatments, including poor compliance, then this may indicate a suboptimal outcome with neuromodulation treatment. Psychological conditions, such as a diagnosis of generalized anxiety disorder or major depressive disorder, that are active or poorly managed, can also potentially lead to poor outcomes with a spinal cord stimulator. Consequently, this is a large reason why, generally, psychological evaluation is required before implanting a patient with a neuromodulation device, as a specialist in the mental health field, generally a psychologist, has the expertise to evaluate for “yellow flags” such as pain catastrophization, and further treatments may be needed for their mental health condition, such as psychotherapy and/or medications.

Certain types of patterns of behavior that should be reviewed include whether the patient has a history of lack of engagement with the treatment plan (failing to attend appointments specific to spinal cord stimulation planning), failing to follow up on agreed recommendations, such as psychotherapy, or attending a pain treatment program without any meaningful engagement. Further types of behavior include dysfunctional coping, avoidance of exercise and activities, inconsistent reports of what the patient states they can do with actual behaviors, restricted participation in daily activities, and poor social and family support. Also, as with any treatment, secondary gain needs to be screened for in all potential spinal cord stimulator patients, including financial or medicolegal reasons.

### 9.3. Clinical and Ethical Considerations: Appropriate Candidate Selection, Informed Consent, and Realistic Expectations

The Neuromodulation Society of Australia and New Zealand (NSANZ) recommends SCS for appropriately selected patients with refractory neuropathic or mixed pain in whom adequate pain relief or functional improvement has not been achieved despite comprehensive multidisciplinary management. The following chronic pain conditions should be considered as treatment options for spinal cord stimulation: persistent spinal pain syndrome, complex regional pain syndrome, peripheral diabetic neuropathy, post-surgical neuropathy, mechanical and nociceptive low back pain, and critical limb ischemia [76].

Informed consent is critical and a pillar of medical ethics and patient care. To facilitate informed decision-making, patient concerns and expectations must be acknowledged, and patients must be provided with clear and accurate information. With spinal cord stimulation, this includes providing patients with what to expect from the procedure, realistic estimates of pain relief, and potential risks and complications. While there are several potential adverse events, as discussed earlier in this chapter, the patient should be made aware of the specific adverse events directly related to spinal cord stimulation, including lead migration, lead fracture, IPG malfunction, and infection. Furthermore, patients should understand that these adverse events can be significant and severe, and this should be explained comprehensively during pre-procedure evaluations. As with any treatment, alternative options should also be discussed, including medications, non-pharmacological treatments such as acupuncture or physical therapy, and interventional pain treatments like epidural steroid injections or radiofrequency ablations [77].

### 9.4. Complications: Epidural Hematoma and Spinal Cord Compression

In rare but serious cases, an epidural hematoma or spinal cord compression situation may arise. In one instance, a 69-year-old man with normal coagulation was undergoing implantation for a spinal cord stimulator. However, once this individual underwent an L1 laminotomy, a large spinal epidural hematoma formed spanning from D4 to L2. This was fixed through prompt surgical intervention [78].

In another rare case, a 58-year-old woman was ready to go for a spinal cord stimulator. However, at T9, an electrode array was implicated in causing the patient to experience spinal stenosis. It was decided then that a T8–T10 spinal cord decompression was needed in which the patient felt immediate partial relief and neurological recovery [79].

Both of these situations are very rare; however, when discussing the safety profile of spinal cord stimulation and possible risks, it is important to mention these cases and potential adverse effects to ensure full transparency.

## 10. Future Directions in Neuromodulation

Future advances in SCS are likely to focus on improving precision, durability, and integration with broader chronic pain care. Next-generation interfaces may include smaller implantable systems, wireless microstimulators, and more advanced bioelectronic platforms designed to reduce hardware burden while improving patient comfort and device flexibility [80]. Although optogenetic neuromodulation remains largely experimental in pain medicine, its ability to control specific neuronal populations with light suggests a possible future direction for highly selective circuit-based therapies [78]. Furthermore, while optogenetics is indeed suggested as a possible future direction, the potential benefits would be much further down the road as the majority of them are speculative.

Closed-loop and adaptive stimulation represent another major direction for the field. Unlike traditional open-loop systems, closed-loop SCS can use real-time feedback, such as evoked compound action potentials (ECAPs), to automatically adjust stimulation intensity and maintain consistent spinal cord activation [79]. As artificial intelligence and machine learning become more integrated into neuromodulation, these systems may eventually help predict patient responses, optimize programming, and personalize stimulation parameters [81].

Personalized waveform programming is also expected to become increasingly important. Current SCS systems already allow clinicians to adjust frequency, pulse width, amplitude, and waveform type; however, future systems may use real-time neurophysiologic monitoring to guide these changes more objectively [79,81]. This could allow treatment to shift away from trial-and-error programming toward data-driven stimulation strategies tailored to each patient’s pain phenotype, neural response, and functional goals.

Finally, future SCS therapy will likely be most effective when integrated into multimodal chronic pain care. Because chronic pain involves sensory, emotional, behavioral, and social factors, neuromodulation should not be viewed as a standalone endpoint. Instead, SCS may be combined with behavioral therapy, physical rehabilitation, digital therapeutics, and emerging biologic or regenerative approaches to improve long-term outcomes [26,64]. Digital therapeutics, including app-based programs and virtual reality interventions, may help reinforce pain education, cognitive–behavioral strategies, activity pacing, and patient engagement outside the clinic [82]. Overall, the future of neuromodulation is moving toward personalized, adaptive, and multidisciplinary care rather than a one-size-fits-all device-based approach.

## 11. Conclusions

SCS has evolved from a therapy primarily focused on suppressing pain symptoms into a sophisticated form of neural circuit modulation. Early systems relied on tonic paresthesia-based stimulation, but newer approaches such as burst stimulation, high-frequency stimulation, closed-loop feedback, and differential target multiplexed stimulation reflect a broader shift toward targeting the underlying neurophysiology of chronic pain [23,65]. Rather than simply masking pain, modern neuromodulation aims to alter spinal and supraspinal pain-processing pathways, improve function, reduce opioid reliance, and enhance quality of life [23,26].

The future of SCS will likely depend on more personalized, predictive, and preventative models of care. Patient selection is increasingly guided by psychological, clinical, anatomic, and neurophysiologic predictors. At the same time, machine learning and closed-loop systems may enable clinicians to identify responders better and optimize stimulation parameters over time [30]. While the tonic and HF10 therapies are more established and have stronger sources of evidence, the DTM and closed-loop methods have made great strides to further support the more personalized neuromodulatory strategies. These advances support a transition from a one-size-fits-all approach toward individualized neuromodulation based on patient-specific pain phenotype, neural response, and functional goals.

Despite these advances, important research gaps remain. Future studies should continue to evaluate long-term durability, mechanisms of loss of efficacy, neuroplasticity markers, and objective biomarkers of treatment response. In addition, SCS should be integrated into broader digital health ecosystems that include behavioral therapy, rehabilitation, remote monitoring, and digital therapeutics [82]. Ultimately, the continued advancement of neuromodulation will depend on combining device innovation with multidisciplinary care to improve long-term outcomes for patients with chronic pain.

## Figures and Tables

**Table 1:** Comparative Evidence from Key RCTs of spinal cord stimulation waveforms.

Waveform	Key RCT and Population	Design	Principal Findings	Evidence (Oxford CEBM)	Limitations	SHAM Control Status
Tonic SCS	PROCESS trial,	Multi-center, parallel group RCT; Tonic SCS with conventional medical management (CMM) vs. CMM alone	6 months, >50% relief in leg pain in 48%, compared to 9% with CMM QoL and treatment satisfaction improved	Level 2	Open-label design, treatment cross- over after 6-month mark,	No; open-label, CMM control
Burst SCS	Sunburst; 100 patients with chronic trunk/limb pain	Multi-center randomized cross-over trial; Burst vs. Tonic	At 24 weeks (around 6 months), Burst was preferred by 70.8% of patients as compared to Tonic SCS	Level 2	Population selected after successful tonic stimulation; no sham control	No; unblinded, tonic SCS control
High Frequency—10 Hz	SENZA-RCT	Multi-center, parallel group RCT; 10 kHz stimulation vs. low-frequency tonic	24-month mark, over 50% back pain relief in 76.5% to 49.3% and for leg pain, 72.9% to 49.3. High-frequency is the preferred treatment	Level 2	Many participants had previous spine surgery or opioid exposure	No; open-label, tonic SCS control
ECAP—Closed Loop	EVOKE	Multicenter RCT; ECAP-controlled closed-loop vs. fixed-output open-loop SCS	12-month mark, over 50% pain reduction without elevated medication in 83.1% closed-loop vs. open-loop with 61%	Level 2	Specialized ECAP may limit the generalized usability of the study	No; double-blinded, SCS active control
DTM SCS	White et al.	Multicenter RCT; DTM stimulation vs. Tonic Stimulation	At 12 months, response rates with DTM (91%) vs. Tonic (25%); Response definition (CLBP ≥ 50% pain relief)	Level 2	Open-label, no sham control, and specific population with neurosurgical concerns	No; open-label, tonic SCS control

## Data Availability

No new data were created or analyzed in this study. Data sharing is not applicable to this article.
